# Construction and Verification of the Molecular Subtype and a Novel Prognostic Signature Based on Inflammatory Response-Related Genes in Uveal Melanoma

**DOI:** 10.3390/jcm12030861

**Published:** 2023-01-21

**Authors:** Feng Zhang, Yan Deng, Dong Wang, Shuai Wang

**Affiliations:** 1 Department of Ophthalmology, Jingzhou Hospital Affiliated to Yangtze University, Jingzhou 434020, China; 2 Department of Hepatobiliary Surgery, Jingzhou Hospital Affiliated to Yangtze University, Jingzhou 434020, China; 3 Department of Thoracic Surgery, Shandong Provincial Hospital Affiliated to Shandong First Medical University, Jinan 250021, China

**Keywords:** uveal melanoma, inflammatory response, immunotyping, prognostic signature, immunotherapy, tumor microenvironment

## Abstract

The significance of inflammation in tumorigenesis and progression has become prominent. This study aimed to construct and validate the molecular subtype and a novel prognostic signature based on inflammatory response-related genes in uveal melanoma (UM). Patients from the TCGA, GSE84976, and GSE22138 UM cohorts were enrolled. According to the consensus cluster analysis, patients were divided into two molecular subtypes, namely IC1 and IC2. Survival curves showed that patients in IC1 had a better prognosis. The IC2 subgroup had higher levels of immune cell infiltration and more enriched immunological pathways. There were statistical differences in the immune-inflammation microenvironment, immune checkpoint genes expression, and drug sensitivity. The prognostic signature constructed based on inflammatory response-related genes exhibited a stable predictive power. Multivariate analysis confirmed that the signature was a prognostic factor independent of clinical characteristics. Functional analyses showed that the high-risk group was associated with immunological response, inflammatory cell activation, and tumor-related signal pathways. The riskscore had a negative relationship with tumor purity and was positively correlated with immune and stromal scores. Furthermore, the prognostic signature could sensitively predict the response to drug treatments. In conclusion, the prognostic signature might aid in stratifying patients at risk premised on the prognosis and immunotherapy sensitivity.

## 1. Introduction

Uveal melanoma (UM) is the most common primary intraocular malignancy in adult humans, accounting for roughly 85–95% of ocular melanoma cases [1,2,3]. UM and cutaneous melanoma stem from melanocytes, but there are differences in biological behavior and treatment outcome [4]. For the primary UM, the main purpose of treatment is to preserve the globe and vision, and prevent distant metastasis. Although lacking a standardized treatment, several treatment approaches such as proton beam radiotherapy, stereotactic radiotherapy, brachytherapy, and local resection could inhibit the local tumor’s uncontrolled growth and even protect the vision of the affected eye [5,6,7]. However, the Collaborative Ocular Melanoma Study (COMS) found no statistically significant difference in melanoma-related mortality between surgical resection and primary brachytherapy. Since then, ocular brachytherapy has emerged as the most common globe-sparing modality and is now the standard gold treatment. Although distant metastases are rare at the initial ocular presentation, 50% of UM patients will develop distant metastases. For metastatic UM, no curative and preventive treatment is available. The main therapies are still based on cutaneous melanoma treatment approaches. Presently, cytotoxic T-lymphocyte-associated antigen-4 inhibitors, PD 1/PD-L1 inhibitors, immune checkpoint inhibitor therapies, cancer vaccines, and cell therapy are being investigated in the treatment utility and made some breakthroughs in treating advanced UM [8,9]. Although some patients have shown good therapeutic responses, the overall results remain limited due to low tumor mutation load, the production of immunosuppressive factors, and new immune checkpoint acquisition [10]. The recent approval of tebentafusp is changing the treatment landscape for patients with metastatic UM [11]. Although the widespread use of early diagnosis and multimodal therapy in clinical practice has significantly improved the prognosis, high mortality affects each UM patient [12]. Growing research has steadily demonstrated the significance of genes and pathways in UM for prediction [13]. However, those biomarkers have proven challenging to quantify precisely due to their high molecular heterogeneity and immunogenicity [14,15]. Hence, it is critical to identify reliable biomarkers associated with the tumor microenvironment to enhance risk prediction ability and individualized guide treatment.

Recently, inflammation, recognized as a prominent characteristic of the tumor, has close associations with tumorigenesis, progression, and treatment resistance [16,17,18]. Tumor-associated inflammation is composed of local inflammation and systemic inflammation. Local tumor inflammation is primarily defined by changes in the tumor microenvironment such as matrix and blood vessel remodeling, as well as immune and inflammatory cell activation, which are related to tumorigenesis, progression, metastasis, and treatment resistance [19,20]. The systemic inflammatory response could result in changes in peripheral blood cell count or protein levels such as lymphocyte, neutrophil, monocyte, platelet, C-reactive protein, and globulin [18]. Systemic inflammatory response biomarkers such as the platelet-to-lymphocyte ratio, lymphocyte-to-monocyte ratio, and neutrophil-to-lymphocyte ratio have been proven to be independent predictors of prognosis in various malignancies [21,22]. Additionally, increasing evidence supported shifting peripheral inflammatory cell counts to create comprehensive prognostic scores for predicting prognosis and drug therapy efficacy. With advancements in research, the roles of inflammation in tumorigenesis have become increasingly prominent. Immune checkpoint therapy is close significant to changes in the immune-inflammation microenvironment. Moreover, patients that exhibit strong tumor inflammatory characteristics have a favorable prognosis. Exploring the relationship between inflammation and tumor might offer optimistic and huge promise in the long run [23,24]. Significantly elevated inflammatory and chemotactic cytokines in UM tissues support the positive associations between inflammation and tumorigenesis [25]. In vivo studies on the aqueous humor have also demonstrated the role of inflammation in UM tumorigenesis [26]. Therefore, exploring the role of inflammatory response in UM was significant in establishing a foundation for using clinical immunosuppressant inhibitors.

The present study aimed to characterize the prognostic significance of inflammatory response-related genes in detail, identify molecular subtypes and establish a prognostic risk signature based on inflammatory response-related genes analysis using comprehensive microarray data from public databases. Moreover, we conducted a systematic analysis of inflammatory response-related molecular subtypes and the prognostic signature in UM patients, which might provide a basis for the clinical prognosis prediction and targeted therapy, chemotherapy, and immunotherapy applications.

## 2. Materials and Methods

### 2.1. Patients and Inflammatory Response-Related Genes Collection

**Figure 1:**
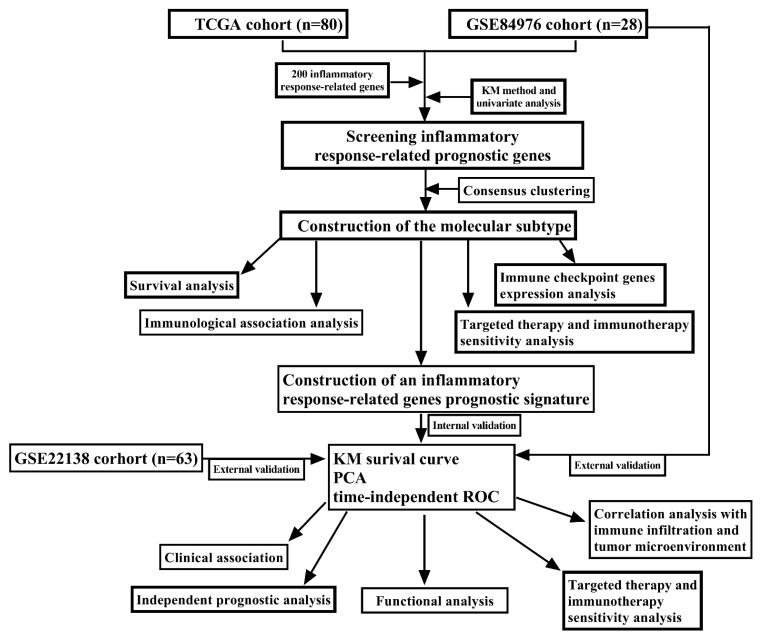
The complete workflow of the analysis in the study.

Clinical data and gene expression profiles of UM patients were obtained from The Cancer Genome Atlas (TCGA) “https://portal.gdc.cancer.gov/ (accessed on 1 October 2022)” and the Gene Expression Omnibus (GEO) “https://www.ncbi.nlm.nih.gov/geo/ (accessed on 1 October 2022)” databases, respectively. The TCGA-UM cohort consisted of 80 patients, whereas 28 UM patients from GSE84976 and 63 UM patients from GSE22138 were, respectively, acquired from the GEO database. Because all the data came from public databases, there was no requirement for a procedure from the ethics committee. Figure 1 depicted the flowchart that summarizes the findings of the study.

The Molecular Signatures database identified 200 genes associated with the inflammatory response, shown in Supplementary Table S1. Subsequently, these genes’ expression levels were, respectively, retrieved from the TCGA and GEO databases.

### 2.2. Molecular Typing Based on Inflammatory Response-Related Genes

Prognostic inflammatory response-related genes were screened using the Kaplan-Meier (KM) method and univariate analysis with significant *p* values less than 0.050. The consensus clustering was conducted in the TCGA cohort using the R “ConsensusClusterPlus” package based on these prognostic genes. According to the cumulative distribution function (CDF), the optimal cluster number and the stable clustering result were obtained. To investigate the universality of the molecular subtype in various cohorts, the GEO cohort was also clustered using the same method. Subsequently, the clinical prognosis was compared between the different molecular subtypes using the R “survival” and “survminer” R packages.

### 2.3. Comparison of Immune Signature, Immune Cell Infiltration Level and Immune-Inflammation Microenvironment between the Molecular Subtypes

According to the single-sample gene-set enrichment analysis (ssGSEA), the enrichment levels of the 29 immune signatures representing diverse immune cell types, functions, and pathways were quantified in each sample [27]. Then, heatmap and boxplot were used to visualize the difference between the molecular subtypes. ESTIMATE [28] analysis was conducted to evaluate the stromal composition (stromal score), immune cell infiltration level (immune score), and tumor purity for each UM sample. These scores were further compared between the different molecular subtypes.

### 2.4. Sensitivity and Efficacy Analysis of Immunotherapy, Targeted Therapy and Chemotherapy between the Molecular Subtypes

Considering the differential expressions of immune signature, immune cell infiltration level and immune-inflammation microenvironment, we also evaluated the expression levels of 47 immune checkpoint genes in various molecular subtypes to evaluate the immunotherapy efficiency. Moreover, the efficacy of targeted therapy and chemotherapy was further compared using the R “pRRophetic” package.

### 2.5. Construction of an Inflammatory Response-Related Genes Risk Signature in the TCGA Cohort

Survival analyses were conducted to screen out genes with prognostic value. To prevent overfitting, LASSO regression analysis was conducted to pick appropriate candidates from those prognostic genes. According to the minimum criteria of the penalty parameter (λ), the ideal genes and their coefficients were obtained to construct a prognostic signature. Finally, a predictive riskscore was calculated by multiplying the sum of their expressions by their coefficient values.

### 2.6. Internal and External Validation of the Inflammatory Response-Related Genes Prognostic Signature

According to the prognostic signature, each participant received a riskscore. Patients were divided into low- and high-risk subgroups premised on the riskscores’ median value. The higher the riskscore, the greater the risk. Following that, the signature’s predictive power was tested both internally and externally in all the cohorts.

The K-M survival curve was plotted to compare the survival difference between high- and low-risk subgroups using the R package “survival”, and a two-sided log-rank test assessed the difference. Subsequently, time-dependent receiver-operating characteristic (ROC) curve analysis using the R “timeROC” package and principal component analysis (PCA) using the “ggplot2” R package were respectively performed to evaluate the prognostic signature’s predictive potential. The area under the receiver-operating characteristic curve (AUC) was computed to assess the sensitivity and specificity. The range of AUC values is 0.5 (no predictive power) to 1 (perfect prediction). Finally, the distribution of patients’ riskscores and the scatter dot plot were visualized to show the detailed associations of the riskscore with death status and survival time.

### 2.7. Independent Prognostic Analysis of the Prognostic Signature

Patients’ riskscores and clinicopathological information were merged to explore the independent prognostic value in all the cohorts. Univariate regression analysis was used to select the parameters with prognostic significance. Multivariate analysis was further done to see if the riskscore could accurately predict patients’ prognoses regardless of their clinicopathological traits.

### 2.8. Functional Analysis

Differentially expressed genes between the low- and high-risk subgroups were identified using the following criteria: an adjusted *p* value < 0.050 and |logFC| > 1. Based on those genes, Gene Ontology (GO) and Kyoto Gene and Genomic Encyclopedia (KEGG) pathway analyses were performed using the R “clusterProfiler” package.

A computational method called gene set enrichment analysis (GSEA) analyzes gene sets to see if a group of genes exhibits a statistically significant difference between two biological states. GSEA was performed to investigate the functional enrichment. Within the “Molecular Signatures Database” of c2.cp.kegg. v6.2. Symbols by GSEA with Java software, potential functions were obtained based on the following criteria: NOM *p* < 0.05 and FDR q < 0.25. 1000 different permutations of the random samples were picked.

### 2.9. Correlations Analysis of the Riskscore with Immune Signature, Immune Cell Infiltration Level, and Tumor Microenvironment-Related Scores

According to the calculated ssGSEA score for each sample, we used a heatmap and boxplot to visualize the differences in immunological signature and immune cell infiltration level between the different categories. Subsequently, the spearman association analysis was performed to examine the relationships between the riskscore and tumor microenvironment-related scores.

### 2.10. Statistical Analysis

Statistical analyses and figure generations were conducted by Perl programming language (version 5.30.2, “http://www.perl.org (accessed on 13 November 2022)”) or the R software (version 4.0.2, “https://www.r-project.org/ (accessed on 13 November 2022)”). The statistical significance was defined as a two-sided *p* < 0.050.

## 3. Results

### 3.1. Molecular Typing Based on Inflammatory Response-Related Genes

**Figure 2:**
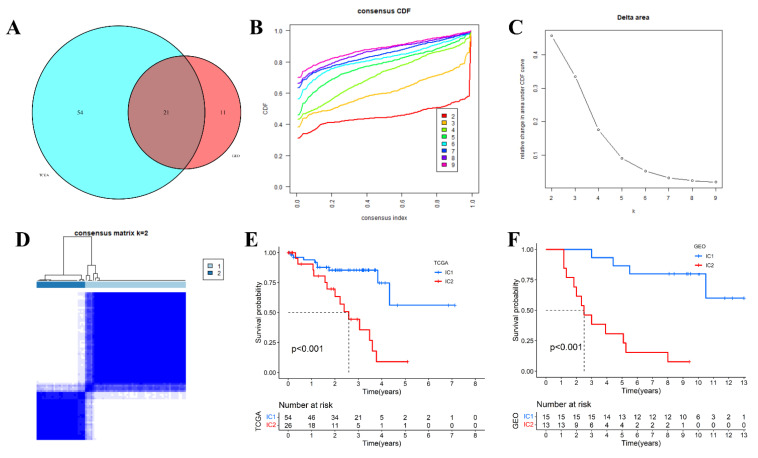
Molecular typing in uveal melanoma. Venn diagram showing the intersected inflammatory response-related genes with prognostic significance in the TCGA and GEO cohort (**A**); The cumulative distribution frequency (CDF) curve of the TCGA-UM cohort samples (**B**); CDF Delta area curve of consensus clustering, which indicates the relative change in area under the CDF curve for each category number k = 2 to k = 10. The vertical axis representing the relative change in area under the CDF curve (**C**). Heatmap of sample clustering when consensus k = 2 in the TCGA cohort (**D**). Kaplan–Meier (KM) curve showing the prognosis of the two subtypes in the TCGA cohort (**E**); KM curve showing the prognosis of the two subtypes in the GEO cohort (**F**).

Survival analysis showed that 75 genes had prognostic significance in the TCGA cohort, while 32 genes were related to prognosis in the GSE84976 cohort. Those prognostic genes from the two cohorts were intersected, yielding 21 intersected genes (Figure 2A). Finally, the 21 inflammatory response-related genes with prognostic value were used for subsequent analyses.

Subsequently, consensus clustering was conducted, and the optimal cluster number was identified based on the CDF in the TCGA cohort. The CDF Delta area curve showed that the clustering results were relatively stable at k = 2 (Figure 2B–D). Next, patients were automatically divided into two inflammatory response-related clusters (inflammatory cluster, IC), which were named IC1 and IC2. The KM survival analysis revealed that the IC1 subgroup’s prognosis was significantly better than the IC2 subgroup’s (Figure 2E). Using the same method, we acquired similar results in the GSE84976 cohort (Supplementary Figure S1 and Figure 2F).

### 3.2. Comparative Analysis of Immune Signature, Immune Cell Infiltration Level and Immune-Inflammation Microenvironment in Different Molecular Subtypes

**Figure 3:**
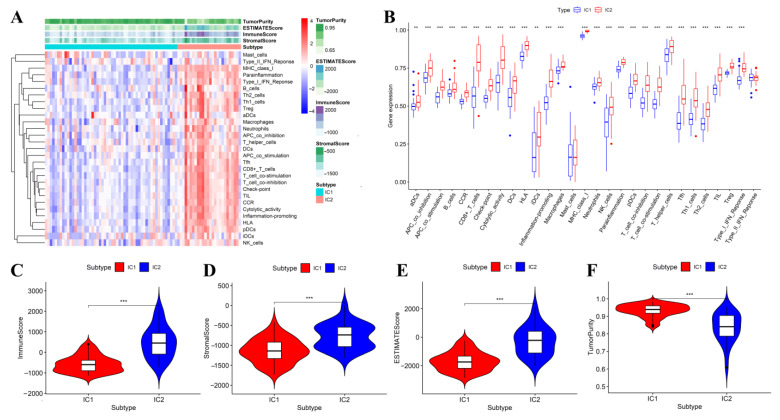
Comparative analysis of the immune signatures, immune cell infiltration level as well as immune-inflammation microenvironment constitution and related scores between the two subtypes in the TCGA cohort. Hierarchical clustering showing the difference in immune cell infiltration and immunological pathways between the two subtypes (**A**); The boxplot showing the differential expressions of 29 immune signatures between the two subtypes (**B**); The violin plot showing the difference in immune score (**C**), stromal score (**D**), estimate score (**E**), and tumor purity (**F**) between the two subtypes. The difference was assessed by a Wilcoxon rank-sum test. *p* < 0.010, and *p* < 0.001 were denoted by “**”, and “***”, respectively.

ssGSEA was applied to quantify the 29 immune-associated signatures in each sample. To explore the potential reasons for poor prognosis in IC2 subtypes, we further compared the difference distribution in different molecular subtypes. Results demonstrated that the IC2 subgroup had higher levels of immune cell infiltration and more enriched immunological signatures (Figure 3A,B). When comparing the immune-inflammation microenvironment between the two IC subgroups, the immune cell infiltration level and stomal score were statistically higher in the IC2 subgroup. In contrast, the tumor purity trended the opposite, with tumor purity increasing from IC2 to IC1 (Figure 3C–F).

### 3.3. Comparison of Immune Checkpoint Genes Expression in Different Subtypes

**Figure 4:**
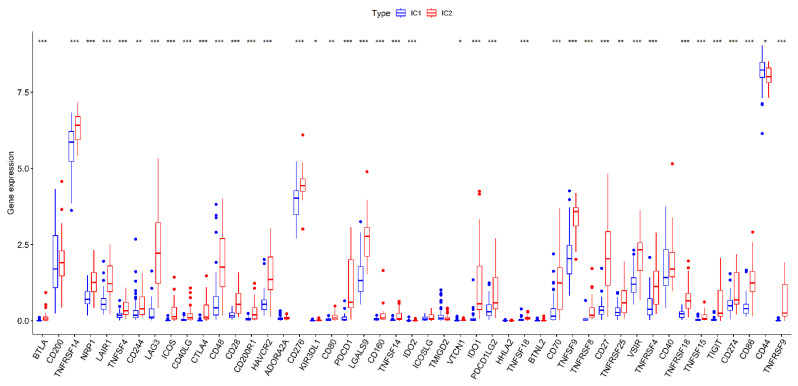
Comparative analysis of immune checkpoint genes expression between the different subtypes in the TCGA cohort samples. *p* < 0.050, *p* < 0.010, and *p* < 0.001 were denoted by “*”, “**”, and “***”, respectively.

As the immune activation, immune cell infiltration level, and immune-inflammation microenvironment play significant roles in immunotherapy, we also compared the expression level of 47 immune checkpoint-related genes or targets for immunotherapy from previous studies. The results confirmed that 40 of the 47 immune checkpoint-related genes or targets (85.11%) expressions differed significantly between the two subtypes. Except for CD44, the other differentially expressed immune checkpoint-related genes were up-regulated in IC2 patients compared with those of IC1 patients (Figure 4).

### 3.4. Differential Analysis of Targeted Therapy and Chemotherapy Sensitivity between Different Molecular Subtypes

**Figure 5:**
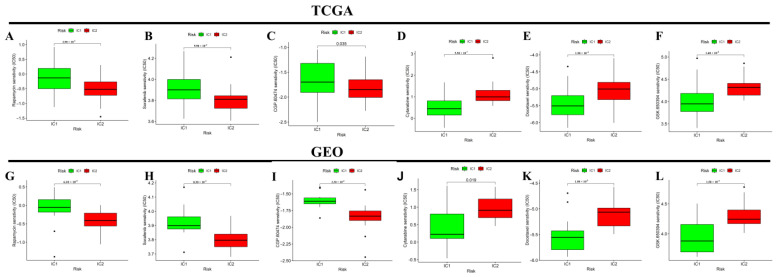
The boxplot showing the difference in targeted therapy and chemotherapy drug sensitivity based on the estimated IC50 between the two subtypes in the TCGA (**A**–**F**) and GEO (**G**–**L**) cohorts. Rapamycin (**A**,**G**); Sorafenib (**B**,**H**); CGP.60474 (**C**,**I**); Cytarabine (**D**,**J**); Docetaxel (**E**,**K**); GSK.650394 (**F**,**L**).

We further compared the differential sensitivity to some targeted therapy and chemotherapy drugs in the two inflammatory subtypes. Compared to the IC2 subgroup, the IC1 subgroup was more sensitive to rapamycin, sorafenib, and CGP.60474; and less sensitive to cytarabine, docetaxel, and GSK.650394 in the TCGA (Figure 5A–F) and GEO (Figure 5G–L) cohorts.

### 3.5. Construction of an Inflammatory Response-Related Genes Prognostic Signature in the TCGA Cohort

**Figure 6:**
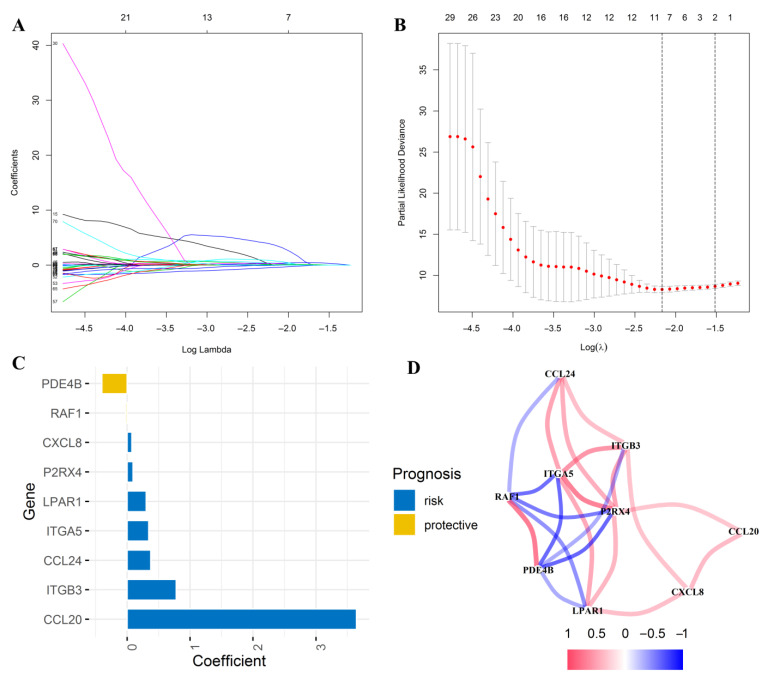
Screening of candidate inflammatory response-related genes in the training group. Selecting the inflammatory response-related potential prognostic genes with a non-zero coefficient corresponding to the same Log Lambda value (**A**); Selection of the optimal variables (Lambda) in the LASSO model (**B**); The histogram exhibited the coefficients of the nine-candidate inflammatory response-related genes (**C**); The correlation network of those candidate genes (**D**).

As mentioned above, we first screened out 75 inflammatory response-related genes significantly related to prognosis according to survival analysis. Subsequently, those prognostic genes were subjected to LASSO regression analysis. Based on the optimal λ value, nine inflammatory response-related genes were retained to establish a prognostic signature (Figure 6A,B). PDE4B and RAF1 were protective variables, while the others were risk factors (Figure 6C). Protein-protein interaction (PPI) network analysis indicated that ITGA5 and P2RX4 were the key genes among those nine genes (Figure 5D). The riskscore for each patient was calculated as follows: Riskscore = (3.644 × CCL20 expression level) + (0.375 × CCL24 expression level) + (0.076 × CXCL8 expression level) + (0.342 × ITGA5 expression level) + (0.779 × ITGB3 expression level) + (0.303 × LPAR1 expression level) + (0.095 × P2RX4 expression level) − (0.403 × PDE4B expression level) − (0.023 × RAF1 expression level).

### 3.6. Internal and External Validation of the Prognostic Signature

**Figure 7:**
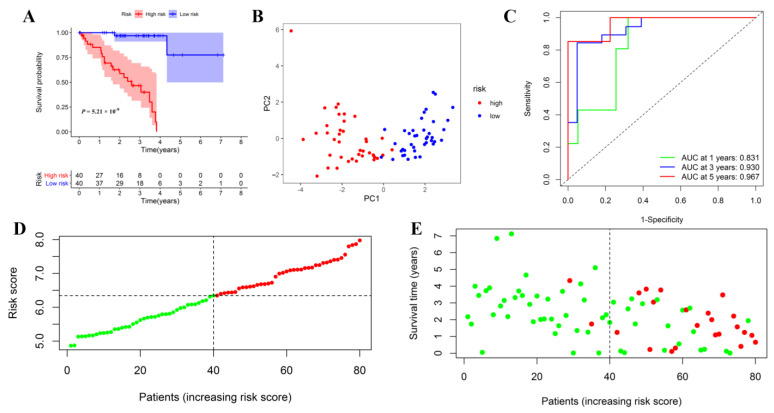
Internal evaluation of the prognostic signature in the training group. Kaplan–Meier (KM) curve for OS of UM patients in the low- and high-risk groups (**A**). Feasibility of PCA-based analysis and judgment models (**B**); Time-dependent ROC curve for 1, 3, and 5-years OS (**C**); Risk curve constructed based on the median of the riskscore (**D**); Survival status of UM patients in the low- and high- risk groups (**E**).

UM patients from the TCGA cohort were assigned into low- or low-risk subgroups based on the median riskscore. The survival curve confirmed that the low-risk subgroup exhibited a significantly better prognosis than the high-risk subgroup (Figure 7A). PCA analysis confirmed that different risk patients were perfectly segmented into two clusters (Figure 7B). Time-dependent ROC curve analysis was used to assess the predictive c significance of the prognostic signature. The AUC was 0.831 at 1-year, 0.930 at 3-year, and 0.967 at 5-year, indicating a perfect prognostic value for predicting survival time (Figure 7C). The riskscore distribution and scatter dot plot confirmed that low-risk patients had less of a chance of dying early than high-risk patients (Figure 7D,E).

**Figure 8:**
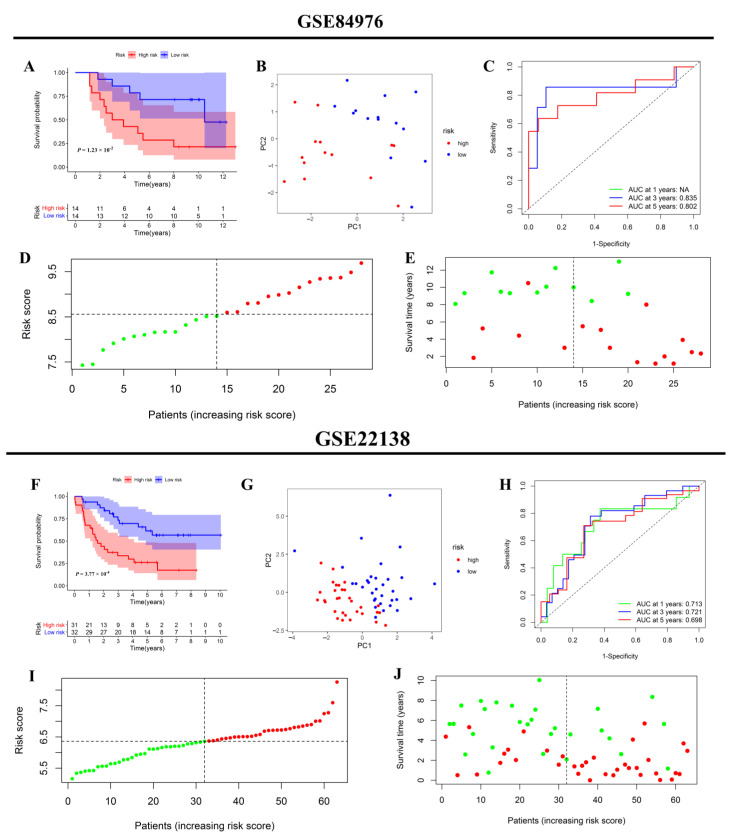
External evaluation of the prognostic signature in the two independent validation groups. GSE84976 (**A**–**E**); GSE22138 (**F**–**J**). KM curves for comparison of the overall survival time and metastasis-free survival time between low- and high-risk groups (**A**,**F**); PCA plot based on the riskscore (**B**,**G**). Time-dependent ROC curves of the riskscore (**C**,**H**). Distribution of riskscores between high- and low-risk groups (**D**,**I**). The distribution of survival status between the low- and high-risk groups (**E**,**J**).

Subsequently, two additional independent validation cohorts have been included to clarify the predictive significance of the prognostic signature. Similarly, each patient obtained a riskscore and was separated into high- or low-risk groups using the aforementioned methods. First, external validation was conducted in the validation group for GSE84976. According to the KM survival curve, patients with a high riskscore experienced considerably shorter survival time than those with low riskscore (Figure 8A). PCA analysis revealed that the patients in distinct risk categories were dispersed in different ways (Figure 8B). Time-dependent ROC curve analysis further confirmed that the prognostic signature had a robust capacity for predicting the prognosis (Figure 8C). The distributions of patients’ riskscores and the scatter chart demonstrated that patients in the high-risk zone died more frequently and had a shorter survival time than those in the low-risk zone (Figure 8D,E). External validation was further performed in another validation cohort for GSE22138. Similar to the previous results, patients in the low-risk group had a lower rate of metastasis than those in the high-risk group. Moreover, the metastasis-free survival time of the high-risk group was also shorter (Figure 8F). As indicated in Figure 7G, PCA analysis demonstrated excellent separation between the high- and low-risk subgroups. Meanwhile, time-dependent ROC curve analysis further validated the robust prediction capacity of the prognostic signature (Figure 8H). The distribution of patients’ riskscores in high- and low-risk groups was ranked in Figure 8I. The scatter plot confirmed that patients in the low-risk group had a lower rate of metastasis and a longer duration without metastasis (Figure 8J).

### 3.7. Correlation Analysis and Independent Prognostic Analysis between the Riskscore and Clinicopathological Features

Correlation analysis between the riskscore and clinicopathological features indicated that riskscore seemed to increase as the tumor progressed, suggesting the prognostic signature could be used to predict cancer development (Table 1). On the other hand, the riskscore was statistically comparable across subgroups categorized by other features except for basal diameter.

**Figure 9:**
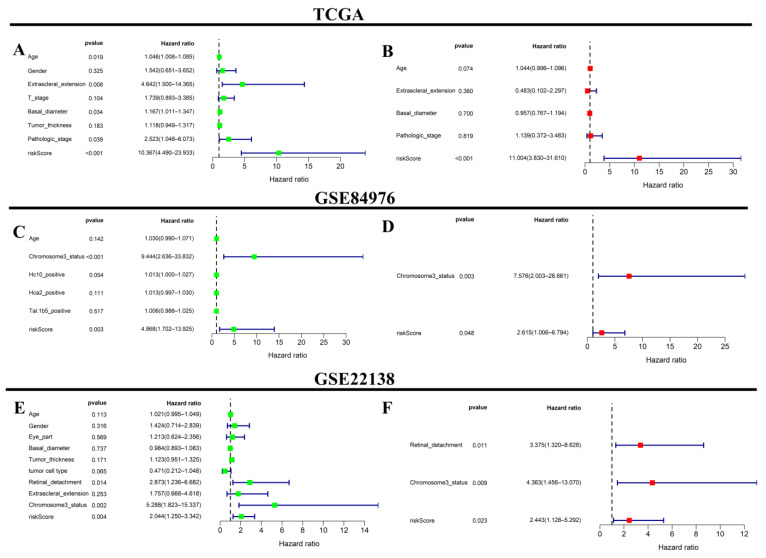
Independent prognostic analysis of the riskscore combined with the clinical characteristics. TCGA (**A**,**B**); GSE84976 (**C**,**D**); GSE22138 (**E**,**F**). Univariate analysis (**A**,**C**,**E**). Multivariate analysis (**B**,**D**,**F**).

Subsequently, to investigate if the predictive signature was independent of clinicopathological characteristics for predicting clinical outcome, univariate and multivariate analyses were conducted after merging the riskscore and clinical variables documented in all cohorts. In the univariate analysis, the riskscore was a prognostic factor (TCGA cohort: HR = 10.376; 95% CI: 4.490–23.933, *p* < 0.001; GSE84976 cohort: HR = 4.868; 95% CI: 1.702–13.925, *p* = 0.003, and GSE22138 cohort: HR = 2.044; 95% CI: 1.250–3.342, *p* = 0.004) (Figure 9A,C,E). After adjusting for other significant factors, multivariate analysis confirmed that the riskscore was an independent prognostic indicator (TCGA cohort: HR = 11.004; 95% CI: 3.830–31.610, *p* < 0.001; GSE84976 cohort: HR = 2.615; 95% CI: 1.006–6.794, *p* = 0.048, and GSE22138 cohort: HR = 2.443; 95% CI: 1.128–5.292, *p* = 0.023) (Figure 9B,D,F).

### 3.8. Functional Analysis

**Figure 10:**
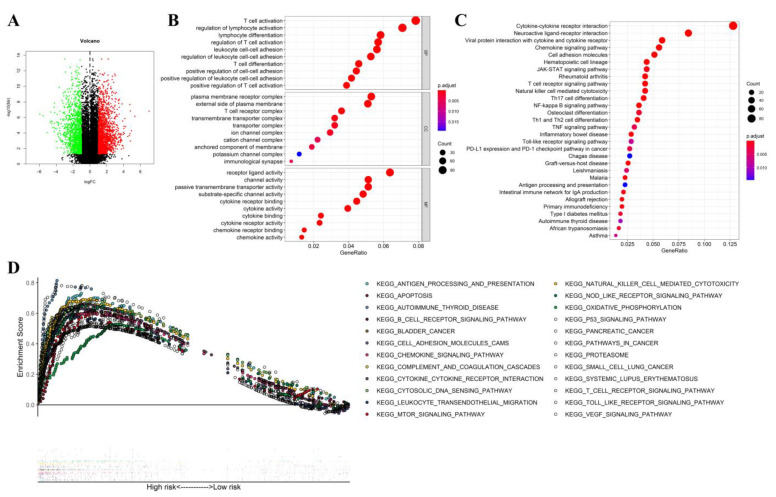
Function enrichment analysis between high- and low-risk UM patients in the training group. Volcano plot for the differentially expressed genes between high- and low-risk groups. Red indicated high expression, and green indicated low expression, Black indicated that these genes showed no significant difference (**A**). Bubble graph for Gene Ontology enrichment analysis (**B**). Bubble graph for the Kyoto Encyclopedia of Genes and Genomes enrichment analysis (**C**). Gene set enrichment analysis (**D**).

As illustrated in the volcano plot, there were 3021 significantly differentially expressed genes between the high- and low-risk subgroups (Figure 10A). Subsequently, GO and KEGG enrichment analyses confirmed that those differentially expressed genes mainly participated in immune regulation, various chemokines and cytokines activation, inflammatory cell chemotaxis, passive transmembrane transporter and channel activity, as well as cytokine-cytokine receptor interaction, etc. (Figure 10B,C).

GSEA analysis confirmed that all enriched gene sets associated with high risk were mainly involved in inflammatory cell activation, chemokine-mediated signaling, immune response, and tumor-associated signaling. Moreover, the more common genes in the high-risk group were linked to tumorigenesis and progress (Figure 10D).

### 3.9. Comparative Analysis of Immune Activation and Immune-Inflammation Microenvironment between the High- and Low-Risk Subgroups

**Figure 11:**
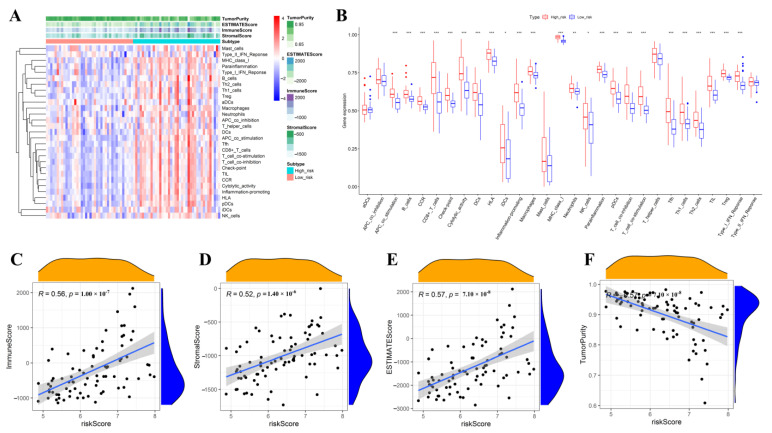
Estimation of the associations of riskscore with immune infiltration estimations and tumor microenvironment in training group. Hierarchical clustering between high- and low-risk groups (**A**). The box plot showed the expression of each sample’s 29 immune signatures between high- and low-risk groups. The difference was assessed by a Wilcoxon rank-sum test (**B**). Scatterplot of associations of riskscore with immune score (**C**), stromal score (**D**), estimate score (**E**), and tumor purity (**F**). *p* < 0.050, *p* < 0.010, and *p* < 0.001 were denoted by “*”, “**”, and “***”, respectively.

The immune activity and immune-inflammation microenvironment-related enrichment scores were further compared between the high- and low-risk subgroups. As shown in Figure 11A,B, the heatmap and boxplot indicated that the high-risk subgroup had much higher levels of immune cell infiltration and a more enriched immune signature. Moreover, correlation analysis showed that riskscore was positively linked with the immune score, stromal score, and estimate score and had a negative association with tumor purity (Figure 11C–F).

### 3.10. Sensitivity and Efficacy Prediction of Immunotherapy and Targeted Therapy

**Figure 12:**
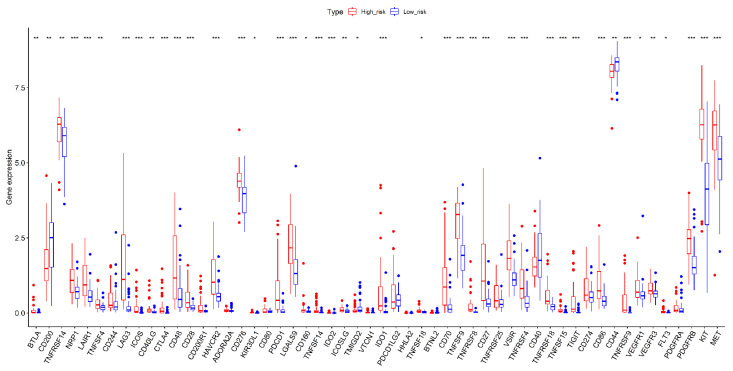
The box plot showing the differences in the expression of immune checkpoint genes and targeted therapeutic target genes between two risk groups in the TCGA cohort samples. *p* < 0.050, *p* < 0.010, and *p* < 0.001 were denoted by “*”, “**”, and “***”, respectively.

Immunotherapy and targeted therapies have gained prominence in treating malignant tumors in recent years. Numerous immunological checkpoints genes and targeted therapy expression levels are essential for personalized treatment, especially in diverse malignancies. Results confirmed that most of the immunological checkpoint and targeted genes significantly differed between the high- and low-risk subgroups. Except for CD44 and CD200, the other differentially expressed genes were up-regulated in the high-risk subgroup compared with those in the low-risk subgroup (Figure 12). Hence, UM patients categorized in high-risk groups might benefit from immunotherapy and targeted therapy.

## 4. Discussion

UM and cutaneous melanoma are both derived from melanocytes, but they behave biologically and respond to treatment in different ways [29]. Patients with skin melanoma have achieved satisfactory results from immunotherapy such as immune checkpoint inhibitors, and significantly improved overall survival time [30]. However, the UM patient has not benefited significantly from immunotherapy [31]. The unsatisfactory results have resulted from the low mutation load or new immune checkpoint acquisition. Moreover, UM Tumor cells’ ceaseless proliferation might disrupt the steady environment within normal eye cells, resulting in an immune-inflammatory microenvironment imbalance [32,33,34]. As is well known, an imbalanced immune-inflammatory microenvironment might promote tumor cell proliferation and mutation, influencing prognosis [35]. Recently, researchers have attempted to treat tumors by regulating the immune-inflammatory microenvironment. It seems that exploring the role of the immune-inflammatory microenvironment in tumorigenesis might shed light on a novel perspective for UM treatment.

In the current study, UM patients were divided into two subtypes after clustering inflammatory response-related genes with prognostic significance. IC1 subtype has a better prognosis. To clarify the root reasons for different prognoses, we performed the ssGSEA to compare the immune signature, immune cell infiltration level and immune-inflammation microenvironment between the different molecular subtypes. We found that the IC2 subgroup had higher levels of immune cell infiltration and more enriched immunological pathways. Moreover, the immune cell activation and immune-inflammation microenvironment-related scores showed significant differences. Those differences might directly influence the tumor progress and immunotherapeutic efficacy. When comparing the response to immunotherapy between the two subgroups, we found that the 39 differentially expressed immune checkpoint-related genes were up-regulated, and CD44 was down-regulated in IC2 UM patients compared with those of IC1 UM patients. These findings suggested that UM patients in the IC2 subtype might benefit more clinically from immunotherapy. Finally, we also found that different subtypes responded differently to traditional targeted therapy and chemotherapy. Compared to the IC2 subgroup, the IC1 subgroup was more sensitive to rapamycin, sorafenib, and CGP.60474; and less sensitive to cytarabine, docetaxel, and GSK.650394. Hence, different immune-inflammation subtypes might potentially offer direction for tailored treatment to UM.

To further simplify clinical study and practice, nine inflammatory response-related core genes were screened out to establish the prognostic signature. Those genes were CCL20, CCL24, CXCL8, ITGA5, ITGB3, LPAR1, P2RX4, PDE4B, and RAF1. CCL20, the major member of the C-C chemokine ligand family, was more abundant in the UM with tumor-infiltrating lymphocytes, which is associated with poor prognosis [36]. Similarly, we also found that high CCL20 levels were significantly related to poor prognosis in UM patients. C-C motif chemokine ligand 24 (CCL24), also known as eotaxin-2, MPIF-2, or Ckβ-6, has been found to aid carcinogenesis and may be employed as a potential biomarker in some malignancies [37,38]. However, the detailed mechanism of CCL24 in UM remains unknown. CXCL8, a typical chemokine of the CXC family, is crucial for the recruitment and activation of neutrophils and granulocytes during inflammation. CXCL8 has been implicated in angiogenesis, metastasis-related tissue remodeling, and tumor cell susceptibility to chemotherapies [39]. Moreover, elevated CXCL8 levels indicate an increased risk of carcinogenesis and a poor prognosis for the disease. Integrin Subunit Alpha 5 (ITGA5), a main member of the integrin alpha chain family, is essential for tumor proliferation, migration, invasion, metastasis, and resistance to chemotherapy [40,41]. Additionally, independent prognosis analysis demonstrated that ITGA5 expression was a risk biomarker in malignancies [42]. Pedro Fuentes et al. demonstrated that ITGB3 is essential for intracellular communication by extracellular vesicles, which is thought to be critical for tumor metastasis [43]. LPAR1 acts in various ways throughout cancer progression and has specialized activities in distinct malignancies. When LPAR1 is overexpressed, it stops the tumor cell growth and migration in gastric cancer [44] but promotes tumor occurrence and progression in the prostate [45] and ovarian cancer [46]. Given the contradictory results in different cancers, our finding involving LPAR1 might provide insights for future research. Consistent with our findings, recent studies have confirmed that P2RX4 could enhance tumor growth and has been considered a potential treatment target [47]. Similar to our finding, Eiji Kashiwagi et al. indicated that down-regulation of PDE4B could activate protein kinase A, resulting in prostate cancer progression [48]. However, Dong Uk Kim et al. stated that PDE4B might regulate the malignant phenotype of colorectal cancer cells and that PDE4B targeting should be pursued aggressively [49]. Studies have verified that RAF1 is carcinogenic in various cancers, but it has also been proven to be a cancer-inhibiting gene in hepatocarcinogenesis [50,51]. Except for CCL20, CCL24, and CXCL8, the genes mentioned above were first discovered to have prognostic value in UM, and the underlying mechanism needs more investigation. Internal validation demonstrated that the established signature could accurately predict prognosis. Subsequently, the predictive value of the prognostic signature was further verified in two independent cohorts from the GEO database. External validation results also demonstrated that the prognostic model could well predict the overall and metastasis-free survival of UM patients. When the associations between riskscore and clinicopathological features were examined, it was discovered that riskscore was positively associated with advanced tumor progression. The explanation might support the finding that an imbalanced immune-inflammatory microenvironment could promote malignant cells’ rapid growth, invasion, and migration, resulting in tumor progression. Predictive analysis showed that riskscore was a prognosis predictor in all the cohorts, independent of all the clinicopathological characteristics. These findings further verified that the prognostic signature possessed powerful therapeutic utility.

Subsequently, functional analysis was performed to clarify the mechanism that the prognostic signature was involved in tumorigenesis and progression. The enrichment analyses indicated that those genes with differential expression mainly participated in immune regulation, activating various chemokines and cytokines, inflammatory cell chemotaxis, passive transmembrane transporter and channel activity, and cytokine-cytokine receptor interaction. GSEA analysis further revealed that the highly expressed genes in the high-risk subgroup were highly implicated in tumor-related signaling pathways and participated in tumorigenesis directly. Moreover, the high-risk group significantly enriched inflammation-related signal pathways such as the B and T cell receptor signaling pathway, cell adhesion molecules cams, as well as natural killer cell-mediated cytotoxicity. According to these results, it is reasonable to hypothesize that those genes affected tumor progression and prognosis by regulating the immune-inflammation microenvironment composition. Consistent with the results above, the high-risk group subgroup had much higher levels of immune cell infiltration and more enriched immunological pathways, demonstrating that the immune-inflammatory environment could affect tumorigenesis and progression. Increased immune cell infiltration and tumor immunological activity in the high-risk subgroup showed that those patients with a high riskscore suffered from an overall impairment of immune functions due to their impaired immune regulation function. Contemporary cancer biology is increasingly focused on cancer cells and their microenvironment, composed of vascular cells, fibroblasts, and inflammatory immune cells. The riskscore was negatively correlated with tumor purity and positively related to the stromal score, immune score, and estimate score. Hence, the findings mentioned above demonstrated that poor survival of UM patients in the high-risk group might be explained by an unbeneficed immune-inflammatory microenvironment and decreased levels of anti-tumor immunity and that high-risk patients could benefit more clinically from immunotherapy. Moreover, the prognostic riskscore might represent each UM patient’s immune-inflammation microenvironment.

Given the close relationships of the riskscore with the immune-inflammation microenvironment, we speculated that patients in distinct risk groups might also respond differently to immunotherapy and targeted therapy. As expected, the expressions of most immune checkpoint-related genes or targets increased in the high-risk subgroup, confirming that UM patients with a higher riskscore might benefit more clinically from immunotherapy and targeted therapy. Hence, the prognostic signature could assess the UM patients’ prognosis and aid clinicians in treatment optimization. However, our study presented serval limitations. First of all, the detailed function mechanisms of inflammatory response-related genes on the tumor have yet to be thoroughly investigated, so we need clinical and basic experiments to confirm the specific mechanism of these genes. Moreover, the research was a retrospective study, and the effectiveness of the constructed molecular subtype and the prognostic signature in clinical practice deserved further exploration by prospective cohort in-depth studies.

## 5. Conclusions

In conclusion, we classified UM patients based on the expressions of the inflammatory response-related genes. Patients in IC1 had a better prognosis, lower levels of immune cell infiltration, and fewer enriched immunological pathways. The immune-inflammation microenvironment, immunotherapy efficacy, and drug sensitivity showed significant differences in different subtypes. The established prognostic signature based on nine inflammatory response-related genes was a reliable tool for predicting the prognosis and the response to immunotherapy and targeted therapy with stable performance in UM patients. These findings might provide insights for further investigation of the inflammatory response-related genes as potential targets in UM. Therefore, the molecular subtype and prognostic signature might assist in stratifying patients at risk based on their prognosis and immunotherapy sensitivity, aiding clinicians in clinical decision-making and individual treatment.

## Figures and Tables

**Table 1:** Correlation analysis of the riskscore with clinicopathological features.

Parameters	Group	Riskscore		
		N	Mean	*p* Value
Age (years)	<60	36	6.158	0.128
	≥60	44	6.441	
Gender	Male	45	6.329	0.853
	Female	35	6.294	
Basal diameter	Low	39	6.101	**0.034**
	High	40	6.490	
Tumor thickness	Low	42	6.360	0.604
	High	38	6.263	
T stage	II	14	5.896	**0.019**
	III	32	6.268	
	IV	34	6.528	
Pathologic stage	II	39	6.111	**0.024**
	III	37	6.448	
	IV	4	7.049	

Notes: The tumor thickness and basal diameter were divided into high and low based on the median value. Bold font represents *p* < 0.050 and the relevant variables are statistically significant.

## Data Availability

Publicly available datasets from the Cancer Genome Atlas “https://portal.gdc.cancer.gov/ (accessed on 1 October 2022)” and the Gene Expression Omnibus (GEO) “https://www.ncbi.nlm.nih.gov/geo/ (accessed on 1 October 2022)” databases were analyzed in this study.

## Supplementary Materials

The following supporting information can be downloaded at: https://www.mdpi.com/article/10.3390/jcm12030861/s1, Supplementary Table S1: A list of 200 genes associated with the inflammatory response. Supplementary Figure S1: The cumulative distribution frequency (CDF) curve of the GSE84976 cohort samples (A); CDF Delta area curve of consensus clustering, indicating the relative change in area under the CDF curve for each category number k = 2 to k = 10. The horizontal axis representing the relative change in area under the CDF curve (B). Heatmap of sample clustering when consensus k = 2 in the GSE84976 cohort (C).

## References

[1] McLaughlin C.C., Wu X.-C., Jemal A., Martin H.J., Roche L.M., Chen V.W. (2005). Incidence of noncutaneous melanomas in the U.S. Cancer.

[2] Aronow M.E., Topham A.K., Singh A.D. (2017). Uveal Melanoma: 5-Year Update on Incidence, Treatment, and Survival (SEER 1973–2013). Ocul. Oncol. Pathol..

[3] Singh A.D., Turell M.E., Topham A.K. (2011). Uveal Melanoma: Trends in Incidence, Treatment, and Survival. Ophthalmology.

[4] Kaliki S., Shields C., Shields J. (2015). Uveal melanoma: Estimating prognosis. Indian J. Ophthalmol..

[5] Bolling J.P., Dagan R., Rutenberg M., Mamalui-Hunter M., Buskirk S.J., Heckman M.G., Hochwald A.P., Slopsema R. (2021). Treatment of Uveal Melanoma with Radioactive Iodine 125 Implant Compared with Proton Beam Radiotherapy. Mayo Clin. Proc. Innov. Qual. Outcomes.

[6] Wei A.Z., Maniar A.B., Carvajal R.D. (2022). New targeted and epigenetic therapeutic strategies for the treatment of uveal melanoma. Cancer Gene Ther..

[7] Weber J.S., D’Angelo S.P., Minor D., Hodi F.S., Gutzmer R., Neyns B., Hoeller C., Khushalani N.I., Miller W.H., Lao C.D. (2015). Nivolumab versus chemotherapy in patients with advanced melanoma who progressed after anti-CTLA-4 treatment (CheckMate 037): A randomised, controlled, open-label, phase 3 trial. Lancet Oncol..

[8] Fu Y., Xiao W., Mao Y. (2022). Recent Advances and Challenges in Uveal Melanoma Immunotherapy. Cancers.

[9] Castet F., Garcia-Mulero S., Sanz-Pamplona R., Cuellar A., Casanovas O., Caminal J.M., Piulats J.M. (2019). Uveal Melanoma, Angiogenesis and Immunotherapy, Is There Any Hope?. Cancers.

[10] Rothermel L.D., Sarnaik A.A., Khushalani N.I., Sondak V.K. (2019). Current Immunotherapy Practices in Melanoma. Surg. Oncol. Clin. North Am..

[11] Martinez-Perez D., Viñal D., Solares I., Espinosa E., Feliu J. (2021). Gp-100 as a Novel Therapeutic Target in Uveal Melanoma. Cancers.

[12] Jager M.J., Shields C.L., Cebulla C.M., Abdel-Rahman M.H., Grossniklaus H.E., Stern M.-H., Carvajal R.D., Belfort R.N., Jia R., Shields J.A. (2020). Uveal melanoma. Nat. Rev. Dis. Prim..

[13] Zhang F., Deng Y., Wang D., Wang S. (2022). Construction and validation of a pyroptosis-related gene signature associated with the tumor microenvironment in uveal melanoma. Sci. Rep..

[14] Smit K.N., Jager M.J., de Klein A., Kiliç E. (2019). Uveal melanoma: Towards a molecular understanding. Prog. Retin. Eye Res..

[15] Fallico M., Raciti G., Longo A., Reibaldi M., Bonfiglio V., Russo A., Caltabiano R., Gattuso G., Falzone L., Avitabile T. (2021). Current molecular and clinical insights into uveal melanoma (Review). Int. J. Oncol..

[16] Lim B., Woodward W.A., Wang X., Reuben J.M., Ueno N.T. (2018). Inflammatory breast cancer biology: The tumour microenvironment is key. Nat. Rev. Cancer.

[17] Amara S., Tiriveedhi V. (2017). Inflammatory role of high salt level in tumor microenvironment (Review). Int. J. Oncol..

[18] Diakos C.I., Charles K.A., McMillan D.C., Clarke S.J. (2014). Cancer-related inflammation and treatment effectiveness. Lancet Oncol..

[19] Chen D.S., Mellman I. (2017). Elements of cancer immunity and the cancer-immune set point. Nature.

[20] Shalapour S., Karin M. (2019). Pas de Deux: Control of Anti-tumor Immunity by Cancer-Associated Inflammation. Immunity.

[21] Deng Y., Zhang F., Yu X., Huo C.-L., Sun Z.-G., Wang S. (2019). Prognostic Value of Preoperative Systemic Inflammatory Biomarkers In Patients With Gallbladder Cancer And The Establishment Of A Nomogram. Cancer Manag. Res..

[22] Wang S., Deng Y., Yu X., Zhang X.-W., Huo C.-L., Sun Z.-G., Chang H. (2021). Prognostic significance of preoperative systemic inflammatory biomarkers in patients with hepatocellular carcinoma after microwave ablation and establishment of a nomogram. Sci. Rep..

[23] Crusz S.M., Balkwill F. (2015). Inflammation and cancer: Advances and new agents. Nat. Rev. Clin. Oncol..

[24] Singh N., Baby D., Rajguru J.P., Patil P.B., Thakkannavar S.S., Pujari V.B. (2019). Inflammation and cancer. Ann. Afr. Med..

[25] Cheng Y., Feng J., Zhu X., Liang J. (2019). Cytokines concentrations in aqueous humor of eyes with uveal melanoma. Medicine.

[26] Midena E., Parrozzani R., Midena G., Trainiti S., Marchione G., Cosmo E., Londei D., Frizziero L. (2020). In vivo intraocular biomarkers. Medicine.

[27] Hänzelmann S., Castelo R., Guinney J. (2013). GSVA: Gene set variation analysis for microarray and RNA-Seq data. BMC Bioinform..

[28] Yoshihara K., Shahmoradgoli M., Martínez E., Vegesna R., Kim H., Torres-Garcia W., Trevino V., Shen H., Laird P.W., Levine D.A. (2013). Inferring tumour purity and stromal and immune cell admixture from expression data. Nat. Commun..

[29] Carvajal R.D., Schwartz G.K., Tezel T., Marr B., Francis J.H., Nathan P.D. (2017). Metastatic disease from uveal melanoma: Treatment options and future prospects. Br. J. Ophthalmol..

[30] Riley R.S., June C.H., Langer R., Mitchell M.J. (2019). Delivery technologies for cancer immunotherapy. Nat. Rev. Drug Discov..

[31] Breazzano M.P., Milam R.W., Batson S.A., Johnson D.B., Daniels A.B. (2017). Immunotherapy for Uveal Melanoma. Int. Ophthalmol. Clin..

[32] Bronkhorst I.H., Jager M.J. (2012). Uveal Melanoma: The Inflammatory Microenvironment. J. Innate Immun..

[33] Bronkhorst I.H.G., Jager M.J. (2012). Inflammation in uveal melanoma. Eye.

[34] Krishna Y., McCarthy C., Kalirai H., Coupland S.E. (2017). Inflammatory cell infiltrates in advanced metastatic uveal melanoma. Hum. Pathol..

[35] Coussens L.M., Werb Z. (2002). Inflammation and cancer. Nature.

[36] Triozzi P.L., Schoenfield L., Plesec T., Saunthararajah Y., Tubbs R.R., Singh A.D. (2014). Molecular profiling of primary uveal melanomas with tumor-infiltrating lymphocytes. Oncoimmunology.

[37] Jin L., Liu W.-R., Tian M.-X., Jiang X.-F., Wang H., Zhou P.-Y., Ding Z.-B., Peng Y.-F., Dai Z., Qiu S.-J. (2016). CCL24 contributes to HCC malignancy via RhoB- VEGFA-VEGFR2 angiogenesis pathway and indicates poor prognosis. Oncotarget.

[38] Lim S.-J. (2021). CCL24 Signaling in the Tumor Microenvironment. Single Mol. Single Cell Seq..

[39] Liu Q., Li A., Tian Y., Wu J.D., Liu Y., Li T., Chen Y., Han X., Wu K. (2016). The CXCL8-CXCR1/2 pathways in cancer. Cytokine Growth Factor Rev..

[40] Wu T., Dai Y. (2016). Tumor microenvironment and therapeutic response. Cancer Lett..

[41] Kuninty P.R., Bansal R., De Geus S.W.L., Mardhian D.F., Schnittert J., van Baarlen J., Storm G., Bijlsma M.F., van Laarhoven H.W., Metselaar J.M. (2019). ITGA5 inhibition in pancreatic stellate cells attenuates desmoplasia and potentiates efficacy of chemotherapy in pancreatic cancer. Sci. Adv..

[42] Zhou C., Shen Y., Wei Z., Shen Z., Tang M., Shen Y., Deng H. (2022). *ITGA5* is an independent prognostic biomarker and potential therapeutic target for laryngeal squamous cell carcinoma. J. Clin. Lab. Anal..

[43] Fuentes P., Sesé M., Guijarro P.J., Emperador M., Sánchez-Redondo S., Peinado H., Hümmer S., Cajal S.R.Y. (2020). ITGB3-mediated uptake of small extracellular vesicles facilitates intercellular communication in breast cancer cells. Nat. Commun..

[44] Ma X., Feng J., Lu M., Tang W., Han J., Luo X., Zhao Q., Yang L. (2019). microRNA-501-5p promotes cell proliferation and migration in gastric cancer by downregulating LPAR1. J. Cell. Biochem..

[45] Shi J., Jiang D., Yang S., Zhang X., Wang J., Liu Y., Sun Y., Lu Y., Yang K. (2020). LPAR1, Correlated with Immune Infiltrates, Is a Potential Prognostic Biomarker in Prostate Cancer. Front. Oncol..

[46] Zhao P., Yun Q., Li A., Li R., Yan Y., Wang Y., Sun H., Damirin A. (2022). LPA3 is a precise therapeutic target and potential biomarker for ovarian cancer. Med. Oncol..

[47] Alharbi A.F., Parrington J. (2021). The role of genetic polymorphisms in endolysosomal ion channels TPC2 and P2RX4 in cancer pathogenesis, prognosis, and diagnosis: A genetic association in the UK Biobank. NPJ Genom. Med..

[48] Kashiwagi E., Shiota M., Yokomizo A., Itsumi M., Inokuchi J., Uchiumi T., Naito S. (2011). Downregulation of phosphodiesterase 4B (PDE4B) activates protein kinase A and contributes to the progression of prostate cancer. Prostate.

[49] Kim D.U., Kwak B., Kim S.-W. (2018). Phosphodiesterase 4B is an effective therapeutic target in colorectal cancer. Biochem. Biophys. Res. Commun..

[50] Ehrenreiter K., Kern F., Velamoor V., Meissl K., Galabova-Kovacs G., Sibilia M., Baccarini M. (2009). Raf-1 Addiction in Ras-Induced Skin Carcinogenesis. Cancer Cell.

[51] Jeric I., Maurer G., Cavallo A.L., Raguz J., Desideri E., Tarkowski B., Parrini M., Fischer I., Zatloukal K., Baccarini M. (2016). A cell-autonomous tumour suppressor role of RAF1 in hepatocarcinogenesis. Nat. Commun..

