# Supplementary material for: Construction and Verification of the Molecular Subtype and a Novel Prognostic Signature Based on Inflammatory Response-Related Genes in Uveal Melanoma
Source: J Clin Med. 2023 Jan 21;12(3):861. doi: 10.3390/jcm12030861 (PMC9918108; doi:10.3390/jcm12030861)
Supplement: Supplementary file 1 [file jcm-12-00861-s001.zip › Supplementary Table S1.pdf]

Supplementary Table S1: Inflammatory response-related genes.

ABCA1	CALCRL	CMKLR1	FPR1	IFNGR2	IRF7	MEP1A	P2RX7	RHOG	SLC7A1
ABI1	CCL17	CSF1	FZD5	IL10	ITGA5	MET	P2RY2	RIPK2	SLC7A2
ACVR1B	CCL2	CSF3	GABBR1	IL10RA	ITGB3	MMP14	PCDH7	RNF144B	SPHK1
ACVR2A	CCL20	CSF3R	GCH1	IL12B	ITGB8	MSR1	PDE4B	ROS1	SRI
ADM	CCL22	CX3CL1	GNA15	IL15	KCNA3	MXD1	PDPN	RTP4	STAB1
ADORA2B	CCL24	CXCL10	GNAI3	IL15RA	KCNJ2	MYC	PIK3R5	SCARF1	TACR1
ADRM1	CCL5	CXCL11	GP1BA	IL18	KCNMB2	NAMPT	PLAUR	SCN1B	TACR3
AHR	CCL7	CXCL6	GPC3	IL18R1	KIF1B	NDP	PROK2	SELE	TAPBP
APLNR	CCR7	CXCL9	GPR132	IL18RAP	KLF6	NFKB1	PSEN1	SELL	TIMP1
AQP9	CCRL2	CXCR6	GPR183	IL1A	LAMP3	NFKBIA	PTAFR	SELENOS	TLR1
ATP2A2	CD14	CYBB	HAS2	IL1B	LCK	NLRP3	PTGER2	SEMA4D	TLR2
ATP2B1	CD40	DCBLD2	HBEGF	IL1R1	LCP2	NMI	PTGER4	SERPINE1	TLR3
ATP2C1	CD48	EBI3	HIF1A	IL2RB	LDLR	NMUR1	PTGIR	SGMS2	TNFAIP6
AXL	CD55	EDN1	HPN	IL4R	LIF	NOD2	PTPRE	SLAMF1	TNFRSF1B
BDKRB1	CD69	EIF2AK2	HRH1	IL6	LPAR1	NPFFR2	PVR	SLC11A2	TNFRSF9
BEST1	CD70	EMP3	ICAM1	IL7R	LTA	OLR1	RAF1	SLC1A2	TNFSF10
BST2	CD82	ADGRE1	ICAM4	CXCL8	LY6E	OPRK1	RASGRP1	SLC28A2	TNFSF15
BTG2	CDKN1A	EREG	ICOSLG	INHBA	LYN	OSM	RELA	SLC31A1	TNFSF9
C3AR1	CHST2	F3	IFITM1	IRAK2	MARCO	OSMR	RGS1	SLC31A2	TPBG
C5AR1	CLEC5A	FFAR2	IFNAR1	IRF1	MEFV	P2RX4	RGS16	SLC4A4	VIP
