# Supplementary figures and images for: Construction and Verification of the Molecular Subtype and a Novel Prognostic Signature Based on Inflammatory Response-Related Genes in Uveal Melanoma
Source: J Clin Med. 2023 Jan 21;12(3):861. doi: 10.3390/jcm12030861 (PMC9918108; doi:10.3390/jcm12030861)

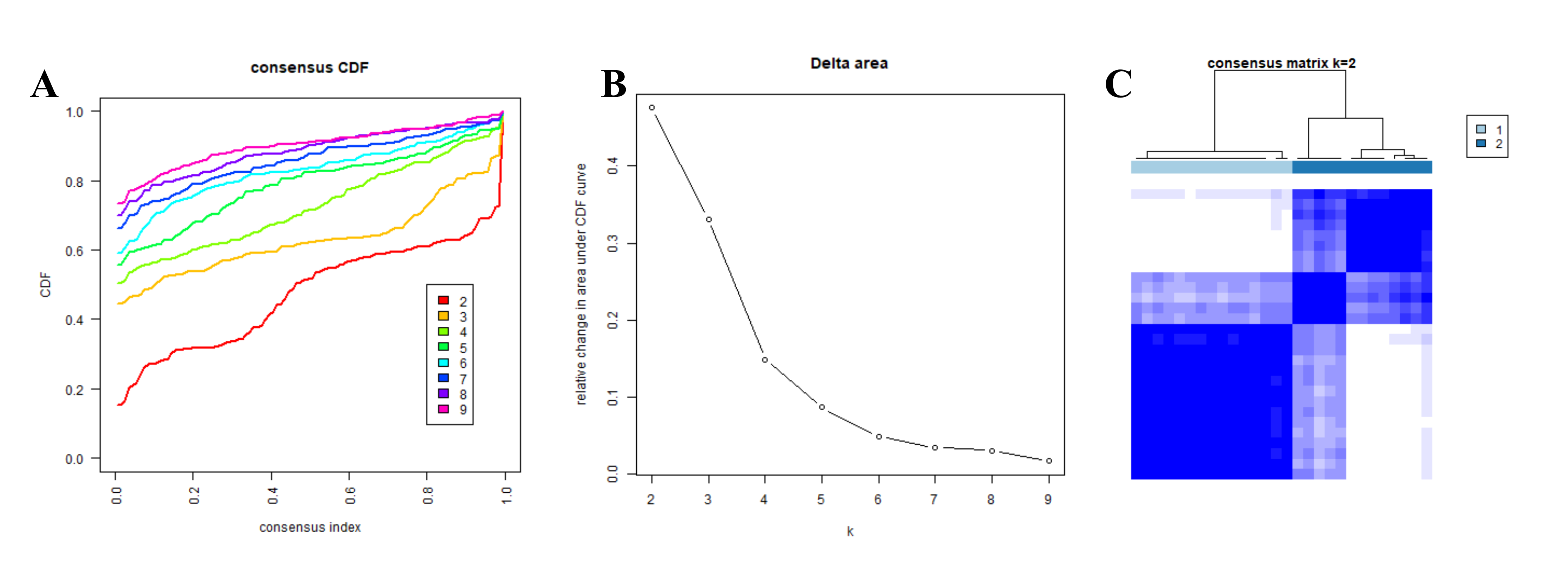

Supplement: Supplementary file 1 [file jcm-12-00861-s001.zip › Supplementary Figure S1.tif]
